# Transcriptomic Characterization of the Human Insular Cortex and Claustrum

**DOI:** 10.3389/fnana.2019.00094

**Published:** 2019-11-27

**Authors:** Christine Ibrahim, Bernard Le Foll, Leon French

**Affiliations:** ^1^Translational Addiction Research Laboratory, Centre for Addiction and Mental Health, Toronto, ON, Canada; ^2^Department of Pharmacology and Toxicology, University of Toronto, Toronto, ON, Canada; ^3^Addictions Division, Centre for Addiction and Mental Health, Toronto, ON, Canada; ^4^Institute of Medical Science, University of Toronto, Toronto, ON, Canada; ^5^Department of Family and Community Medicine, University of Toronto, Toronto, ON, Canada; ^6^Division of Brain and Therapeutics, Department of Psychiatry, University of Toronto, Toronto, ON, Canada; ^7^Campbell Family Mental Health Research Institute, Centre for Addiction and Mental Health, Toronto, ON, Canada; ^8^Krembil Centre for Neuroinformatics, Centre for Addiction and Mental Health, Toronto, ON, Canada

**Keywords:** transcriptomics, insula, claustrum, addiction, depression, epilepsy, dopamine, macroautophagy

## Abstract

The insular cortex has been linked to a multitude of functions. In contrast, the nearby claustrum is a densely connected subcortical region with unclear function. To view the insula-claustrum region from the molecular perspective we analyzed the transcriptomic profile of these areas in six adult and four fetal human brains. We identified marker genes with specific expression and performed transcriptome-wide tests for enrichment of biological processes, molecular functions, and cellular components. In addition, specific insular and claustral expression of genes pertaining to diseases, addiction, and depression was tested. At the anatomical level, we used brain-wide analyses to determine the specificity of our results and to determine the transcriptomic similarity of the insula-claustrum region. We found *UCMA* to be the most significantly enriched gene in the insular cortex and confirmed specific expression of *NR4A2*, *NTNG2*, and *LXN* in the claustrum. Furthermore, the insula was found to have enriched expression of genes associated with mood disorders, learning, cardiac muscle contraction, oxygen transport, glutamate and dopamine signaling. Specific expression in the claustrum was enriched for genes pertaining to human immunodeficiency virus (HIV), severe intellectual disability, epileptic encephalopathy, intracellular transport, spine development, and macroautophagy. We tested for enrichment of genes related to addiction and depression, but they were generally not highly specific to the insula-claustrum region. Exceptions include high insular expression of genes linked to cocaine abuse and genes associated with ever smoking in the claustrum. Brain-wide, we find that markers of the adult claustrum are most specifically expressed in the fetal and adult insula. Altogether, our results provide a novel molecular perspective on the unique properties of the insula and claustrum.

## Introduction

The insular cortex (IC), part of the cerebral cortex, is commonly divided into anterior and posterior parts which differ in functional connectivity, as well as in cytoarchitecture (Mesulam and Mufson, 1982; Deen et al., 2011; Kelly et al., 2012). It is also subdivided into subregions known as granular, dysgranular, and agranular (Paxinos and Watson, 1986). The former terminology is commonly used when referring to the human IC, while the latter is used in rodents. The insula as a whole has major bidirectional connections to several regions such as the anterior cingulate cortex, orbitofrontal cortex, supplementary motor areas, amygdala, etc. (Augustine, 1996; Flynn, 1999). Along with these connections, the IC has a multitude of functions, most notably its involvement in conscious urges, homeostasis, interoception, decision making, anxiety and cognition (Craig, 2002; Paulus and Stein, 2006; Chang et al., 2013; Droutman et al., 2015a).

The claustrum, whose name means “hidden away,” is a thin bilateral brain region made of gray matter that is embedded in the white matter beneath the IC and above the putamen (Crick and Koch, 2005). By volume, it is the most heavily connected structure in the brain (Torgerson et al., 2015). Mouse tract tracing studies have found that it is connected to almost every region in the cortex (Wang et al., 2017). However, due to its small size and location, its functional roles are not entirely understood. It has been postulated that the claustrum is an integral area for bringing together information within and across sensory and motor modalities to form one joint experience in consciousness (Crick and Koch, 2005).

The establishment of atlases that assay genome-wide gene expression in the human brain by the Allen Institute for Brain Science and their collaborators allows for in-depth analysis of over 200 regions. These comprehensive atlases include the long and short insular gyrus (i.e., posterior and anterior IC, respectively) and the claustrum (Hawrylycz et al., 2012; Miller et al., 2014a). To date, gene expression studies in these regions have been limited in the number of genes investigated and to rodent brains (D’Souza et al., 2008; Mathur et al., 2009; Dillingham et al., 2017; Wang et al., 2017). An exception is a four-gene study that examined the monkey claustrum to find neocortical similarities (Watakabe et al., 2014). A detailed analysis of the transcriptomic profile of the insula and claustrum in the human brain would be of value to further solidify what is known of these areas, as well as to provide new insights.

In this study, using human transcriptomic data, we identified genes with specific expression in the adult and fetal IC and claustrum. In addition, we also tested if genes associated with particular functions and diseases are uniquely expressed in these neighboring regions. Given past associations of these regions, we undertook addiction and depression focused analyses (Naqvi and Bechara, 2010; Sliz and Hayley, 2012; Bernstein et al., 2016; Gogolla, 2017). Due to the neurodevelopmental links of these adjacent regions, we compared their transcriptomic profiles to determine overlapping patterns (Puelles, 2014; Watson and Puelles, 2017; Binks et al., 2019).

## Materials and Methods

Broady, we extended methods that have been previously used for the characterization of the habenula (Le Foll and French, 2018).

### Adult Human Brain Gene Expression Data

Transcriptomic data that comprehensively assays the adult human brain was obtained from the Allen Human Brain Atlas (Hawrylycz et al., 2012). All six brains assayed in the Atlas contained insular gyri and claustrum samples (five males one female; aged 24 to 57 years old). As documented by the Allen Institute, postmortem blood was tested for the presence of therapeutic drugs and drugs with abuse potential. Caffeine (all 6 donors), theobromine (3 donors), atropine (3 donors), lidocaine (1 donor), monoethylglycinexylidide (1 donor), and ibuprofen (1 donor) were detected at levels that are not considered toxicologically significant. The Allen Institute also assayed RNA integrity (RIN), which, across the six brains, ranged from 6.3 to 7.5 in the frontal poles, 5.8–7.1 occipital poles, 6.9–8.6 in the cerebellum, and 5.6–7.3 in the brainstem. In total, 3,702 spatially resolved gene expression profiles were used, providing expression information for 232 unique named brain regions. The 58,692 microarray probes were filtered to the 48,170 that mapped to the 20,778 gene symbols in the Allen annotations. Details of the procedures used by the Allen Institute researchers to annotate and normalize the data are available at the Allen Human Brain Atlas website^1^.

### Prenatal Human Gene Expression Data

We additionally used the transcriptomic atlas for the normal mid-gestational human brain that was created by the BrainSpan consortium (Miller et al., 2014a). As noted by Brainspan Consortium, RIN values averaged 6.3. The dorsal claustrum, dysgranular and granular IC were acquired in all four of the prenatal specimens used for this atlas (15–21 postconception weeks, 3 females and one male). The ventral claustrum was assayed in three of the specimens (1 male and 2 females). The specimens passed several exclusion criteria and no neuropathological defects were found by the consortium. Data from the four specimens contained 1,203 spatially resolved gene expression samples, providing transcriptomic data for 516 unique named brain regions. The same custom microarrays that were used for the adult atlas were used to profile expression. Details of the procedures used by the Allen Institute researchers are available at the BrainSpan website^2^.

### Region-Specific Expression Analysis

Mirroring our previous work that targeted the habenula (Le Foll and French, 2018), we used the limma software package to detect probes that are specifically expressed in the claustrum and insula (Ritchie et al., 2015). In the adult data, those regions are: claustrum, short insular gyri, and long insular gyri. In the fetal brain, we used data from the agranular IC (area Iag), dysgranular IC, granular IC, dorsal claustrum, and ventral claustrum. Data from the dorsal and ventral claustrum were combined to form a single claustrum grouping for comparison with the adult data. Two samples from the agranular IC (area Iag) were grouped with the dysgranular IC to simplify analysis. Unlike the adult dataset, the fetal data has fine dissections of cortical zones and layers. We grouped these finer samples into their enclosing cortical regions. This grouping reduces the 516 unique named brain regions in the fetal data to 283. For each microarray probe, linear models were fit with coefficients for donor and region of interest. In other words, expression of a given probe across the expression measurements (adult:3,702, fetal:1,203) was modeled with variables indicating the donor and if the sampled region was the specific region of interest or not. Separate analyses were undertaken for each region of interest instead of fitting a linear model with coefficients for every brain region. The empirical Bayes moderation method implemented in limma was used to calculate moderated t statistics and corresponding *p*-values (Ritchie et al., 2015). We used the Benjamini-Hochberg false discovery rate (FDR) procedure to correct for the many tested probes (Benjamini and Hochberg, 1995). To summarize the probe level statistics, we used the probe to gene mappings provided by the Allen Institute for the fetal data (Hawrylycz et al., 2012; Miller et al., 2014b). For a given gene, we summarized the region-specific expression results by choosing the probe with the lowest *p*-value to represent the gene. The threshold for significance was set to *p*_FDR_ < 0.05 at the probe level and then applied to the minimum *p*-values at the gene level.

### Gene Set Enrichment Analysis

For a given region of interest, *p*-values were combined with the direction of effect for the 20,778 genes (signed *p*-values). The resulting ranking starts with the gene with the most significant specific expression to the gene with the most significant depleted expression in the region. This ranking allowed us to test if the genes that were specifically expressed in the claustrum or insula are enriched for a given gene set using the area under the receiver operator curve (AUROC) statistic. The AUROC for a set of genes is equivalent to the probability that a gene associated with that set will be found first in the genome-wide ranking compared to all other genes. In this context, AUROC > 0.5 for a gene set means that these genes are more likely to have higher expression in a region of interest. In contrast, an AUROC < 0.5 marks a bias toward lower expression. Given our focus on up-regulation, we only tested for AUROC values above 0.5. AUROC values were calculated with the tmod analysis package in R (Weiner and Domaszewska, 2016). The Mann–Whitney *U* test was used to determine statistical significance (one-sided). We again used the FDR procedure to adjust for the many tested gene sets.

We also use specificity tests to determine if the enriched gene sets are not representing broad differences that are up-regulated in many other brain regions. For example, insula specific gene sets may simply represent neocortex specific expression. To assess specificity, we ran the region-specific expression and gene set enrichment procedures for 283 fetal and 232 named brain regions in the expression datasets. For each gene set, we counted the number of regions with an AUROC value higher than the region of interest. Gene sets that have the highest specificity were considered to characterize the region of interest uniquely.

### Gene Ontology Gene Sets

The Gene Ontology (GO) consortium annotates genes to biological processes, molecular functions, and cellular locations to formally model biological systems using controlled vocabularies (Ashburner et al., 2000). The GO database was accessed through the GO.db and org.Hs.eg.db packages in R (Carlson, 2017a, b). Annotations were dated October 10, 2018. We limited our tests to GO groups containing between 10 and 200 genes, after filtering for genes contained in the Allen microarray data (7,089 GO groups annotating 15,017 genes).

### Estimation of Cell-Type Proportions

The markerGeneProfile R package was used to estimate cell type proportions from the transcriptomic data (Ogan et al., 2017). This method uses the first principal component obtained from a set of cell-type specific markers to estimate the relative abundance of a cell type. For cell-type markers, we used genes from a study of healthy human temporal cortex tissue (Darmanis et al., 2015). This study provided the top 21 most enriched genes for astrocytes, neurons, oligodendrocytes, oligodendrocyte precursors, microglia and endothelial cell-types [Supplementary Table S3; (Darmanis et al., 2015)]. The default parameters for the mgpEstimate function were used. Proportions were estimated separately for each donor brain. Within a brain, proportions were mean averaged across multiple samples for each brain region. Regional proportions for each cell-type were then scaled and then mean averaged across the brains. Ranks were then computed across all regions for each cell-type to provide relative rankings of estimated proportions.

### Disease-Associated Gene Sets

Human disease-associated gene sets were obtained from the DisGeNET database, which integrates disease-gene links from several sources (Piñero et al., 2017). The curated gene-disease association file was downloaded in May 2018. Similar to the GO sets, we used disease-associated gene sets with 10 to 200 genes (1,848 disease-associated gene sets covering 5,865 unique genes). The gene set named “severe mental retardation (I.Q. 20–34)” was renamed to “severe intellectual disability (I.Q. 20–34)” to reflect newer terminology.

To complement the heterogeneous data that is used to build the DisGeNET, we used genes from large genetic studies focused on addiction or depression. For lifetime cannabis use associations, we used the seven genes harboring the genome-wide significant loci from Table 1 in Pasman et al. (2018). Nine genes near the 14 genome-wide loci associated with alcohol consumption were obtained from Clarke et al. (2017). Genes associated with several smoking-related measures were identified in Minicã et al. (2017). Four genes linked to substance use disorder and five associated with opioid use disorder were obtained from a review of genetic studies by Jensen (2016). For depression, we used the 70 genes that neighbor the 44 significant loci identified in the largest genetic study of major depression to date [Table 2 in Wray et al. (2018)]. We also used a more recent list of 269 genes from a large study of depression [Supplementary Table S9 in Howard et al. (2019)].

### Transcriptomic Similarities Between the Claustral and Insular Regions

Complete expression profiles (58,692 probes) were clustered into two dimensional space with the Uniform Manifold Approximation and Projection (UMAP) method (McInnes et al., 2018). This general-purpose dimensionality reduction method is built from principles in Riemannian geometry and algebraic topology. The R implementation was used with default configuration parameters.

We computed the transcriptional similarity between the adult claustrum and all other regions using the AUROC method. To provide a finer resolution, we did not collapse the developing cortical zones. Instead of all genome-wide significant genes, only the top 20 genes for the claustrum were used to provide a specific signal.

## Results

### Insular Cortex

#### Long Insular Gyri and Granular Insular Cortex

We first investigated up-regulated expression in the long insular gyri in adult Atlas. In total, there were 48,165 tested microarray probes, which mapped to 20,778 genes. Of these genes, 1,273 were significantly up-regulated in the 22 long insular gyri samples in comparison to the rest of the brain (*p*_FDR_ < 0.05). The top 20 genes are presented in Table 1, and the full probe level results are in Supplementary Table S1. We also tested the human fetal brain and found 733 significantly enriched genes in the 19 granular IC samples. At this mid-gestational stage, corticogenesis is well underway as nine zones can be delineated in the developing neocortex (Bystron et al., 2008; Miller et al., 2014a). Overlap was observed between the adult and fetal results with 160 intersecting genes (hypergeometric test, *p* < 10^–46^). However, no overlap was found between the top 20 lists, but we note that the majority of genes are driven by more than one probe.

**TABLE 1:** Top 20 enriched genes in the adult long insular gyri.

**Gene**	**Name**	**Significant**	***p*-value**
**Symbol**		**probes**	
UCMA	Upper zone of growth plate and cartilage matrix associated	2	4.63E-45
NAA11	N(alpha)-acetyltransferase 11, NatA catalytic subunit	1	9.89E-17
MMP3	Matrix metallopeptidase 3	2	1.2E-16
MUC19	Mucin 19, oligomeric	1	3.27E-09
NTNG2	Netrin G2	3	3.44E-09
DCSTAMP	Dendrocyte expressed seven transmembrane protein	1	4.99E-09
LAIR2	Leukocyte associated immunoglobulin like receptor 2	2	5.28E-09
PGA3	Pepsinogen 3, group I (pepsinogen A)	2	1.44E-08
SMIM32	Small integral membrane protein 32	2	1.68E-08
GYPE	Glycophorin E (MNS blood group)	1	3.42E-08
LOC100129291	Chromosome X open reading frame 49 pseudogene	2	4.32E-08
DUSP13	Dual specificity phosphatase 13	2	6.67E-08
TMEM233	Transmembrane protein 233	2	7.01E-08
OLFML2B	Olfactomedin like 2B	1	9.83E-08
NPPA	Natriuretic peptide A	2	9.83E-08
LYZL4	Lysozyme like 4	2	9.86E-08
FAM217A	Family with sequence similarity 217 member A	1	1.55E-07
HABP2	Hyaluronan binding protein 2	1	1.8E-07
TEPP	Testis, prostate and placenta expressed	2	2.19E-07
ANXA8	Annexin A8	1	2.31E-07

#### Short Insular Gyri and Dysgranular Insular Cortex

With regards to the 22 adult short insular gyri samples, 2,697 genes were significantly up-regulated (*p*_FDR_ < 0.05). The top 20 genes are shown in Table 2 (probe level results in Supplementary Table S2). In the fetal brain data, there were 881 enriched genes in the 22 dysgranular IC samples. Between the two, there were 407 intersecting genes (hypergeometric test, *p* < 10^–134^) and two genes overlap within the top 20 lists (*TFAP2D* and *SLN*).

**TABLE 2:** Top 20 enriched genes in the adult short insular gyri.

**Gene**	**Name**	**Significant**	***p*-value**
**Symbol**		**probes**	
UCMA	Upper zone of growth plate and cartilage matrix associated	2	3.12E-33
KRT1	Keratin 1	1	3.14E-17
LAIR2	Leukocyte associated immunoglobulin like receptor 2	2	8.18E-16
GSG1	Germ cell associated 1	1	2.1E-14
LYZL4	Lysozyme like 4	2	3.27E-14
MS4A8	Membrane spanning 4-domains A8	1	2.59E-13
DUSP13	Dual specificity phosphatase 13	2	3.19E-13
TFAP2D	Transcription factor AP-2 delta	2	4.56E-13
GYPE	Glycophorin E (MNS blood group)	1	3.22E-12
PGA3	Pepsinogen 3, group I (pepsinogen A)	2	6.13E-12
SMYD1	SET and MYND domain containing 1	2	2.89E-11
SCARA5	Scavenger receptor class A member 5	1	3.27E-11
DCSTAMP	Dendrocyte expressed seven transmembrane protein	2	3.92E-11
KLK5	Kallikrein related peptidase 5	2	4.35E-11
GYPB	Glycophorin B (MNS blood group)	1	4.73E-11
FREM3	FRAS1 related extracellular matrix 3	2	5.69E-11
TWIST2	Twist family bHLH transcription factor 2	1	6.21E-11
SLN	Sarcolipin	2	6.73E-11
NTNG2	Netrin G2	3	7.29E-11
RS1	Retinoschisin 1	2	1.13E-10

#### Genes Upregulated Across the Insular Cortex

**FIGURE 1:**
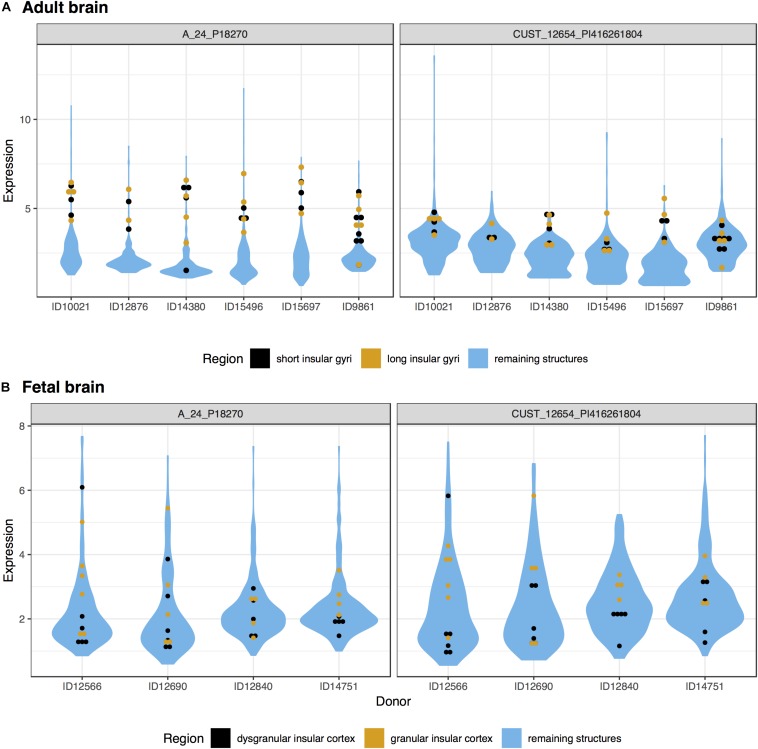
Plots of *UCMA* expression in the adult **(A)** and fetal **(B)** brains. Expression (log2 intensity) is plotted on the *y*-axis for each of the two probes for *UCMA*. Donor identification numbers are marked on the *x*-axis. Expression in the short insular gyri and dysgranular insular cortex is marked in black with orange marking the long and granular divisions. Expression across the remaining brain regions is shown in blue violin plots.

In the adult data, many genes had high expression in both the long and short insular gyri. Of the significantly up-regulated genes, 766 intersected between the two subregions of the IC (hypergeometric test, *p* < 10^–50^). Within the top 20 gene lists, 8 genes were in common (*UCMA, NTNG2, DCSTAMP, LAIR2, PGA3, GYPE, DUSP13*, and *LYZL4; p* < 10^–19^). *UCMA* was the most significantly enriched gene in both regions (Figure 1). In the fetal data, there were 213 genes that are significantly up-regulated in both the granular and dysgranular IC (hypergeometric test, *p* < 10^–50^). Of these, 11 genes were found in both of the top 20 lists (*MIR133A1, KIF16B, TMEM244, NR4A2, C1orf115, BHMT, TIPARP, PTPRK, RSPO2, RASGEF1C*, and *GRP; p* < 10^–29^).

#### Gene Ontology Enrichment

In the adult data, after correcting for multiple comparisons, 252 GO groups were found to be significantly enriched in the long insular gyri and 216 in the short insular gyri (full results in Supplementary Tables S3, S4). With regards to the fetal data, 293 groups were significantly enriched in the dysgranular IC and 90 groups in the granular IC.

**FIGURE 2:**
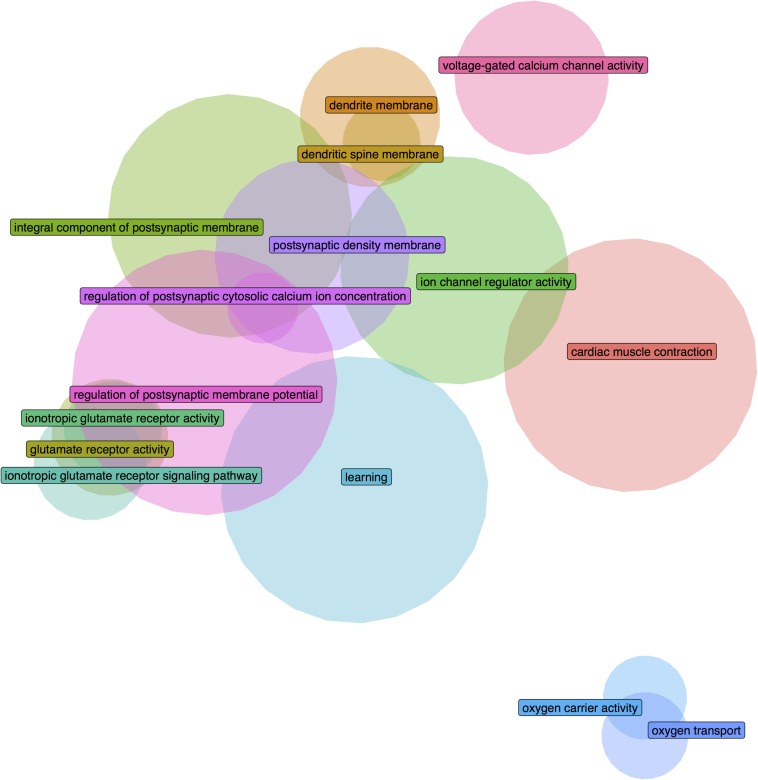
Euler diagram demonstrating gene overlaps between the significantly enriched GO groups in the adult long insular gyri that are also validated in the fetal granular insular cortex.

We further filtered the results to only include groups that have a higher AUROC statistic in one or no other regions of the 232 tested brain-wide (specificity rank of ≤1). With this criterion, 26 GO groups remained in the adult long insular gyri, 7 in the adult short insular gyri, 88 in the fetal dysgranular IC and 4 in the fetal granular IC. Of the 26 groups in the adult long insular gyri that survived the cutoff, 15 are also significantly up-regulated in the fetal granular IC (*p*_uncorrected_ < 0.05). These GO groups mainly pertain to glutamate activity and oxygen transport (Table 3 and Figure 2). Of the seven groups that survived the cutoff filter in the adult short insular gyri, “dopamine receptor signaling pathway” and “nucleotide-sugar metabolic process” are enriched in the fetal dysgranular IC (*p*_uncorrected_ < 0.05) (Table 4).

**TABLE 3:** Enriched GO groups in the adult long insular gyri that are also enriched in the fetal granular insular cortex.

**Name**	**Genes**	**AUROC**	**Specificity rank**	***p*-value_FDR_**	**Fetal AUROC**	**Fetal *p*-value**
Integral component of postsynaptic membrane	117	0.712	0	1.82E-12	0.635	2.49E-07
Regulation of postsynaptic membrane potential	139	0.688	1	8.64E-12	0.625	2.03E-07
Postsynaptic density membrane	75	0.729	1	1.75E-09	0.685	1.52E-08
Learning	141	0.652	1	6.13E-08	0.543	0.0389
Ion channel regulator activity	103	0.648	1	1.53E-05	0.599	2.59E-04
Glutamate receptor activity	27	0.77	0	6.93E-05	0.674	8.76E-04
Ionotropic glutamate receptor activity	19	0.799	1	2.80E-04	0.661	0.00748
Dendrite membrane	39	0.703	1	4.68E-04	0.635	0.00177
Ionotropic glutamate receptor signaling pathway	25	0.751	0	5.37E-04	0.623	0.0163
Oxygen carrier activity	14	0.764	1	0.0157	0.793	7.34E-05
Oxygen transport	15	0.74	1	0.0246	0.788	5.64E-05
Cardiac muscle contraction	127	0.583	1	0.0253	0.571	0.00302
Voltage-gated calcium channel activity	47	0.631	1	0.0332	0.586	0.0203
Regulation of postsynaptic cytosolic calcium ion concentration	10	0.769	1	0.0481	0.719	0.00831
Dendritic spine membrane	12	0.743	1	0.0494	0.777	4.51E-04

**TABLE 4:** Enriched GO groups in the adult short insular gyri that are also enriched in the fetal dysgranular insular cortex.

**Name**	**Genes**	**AUROC**	**Specificity rank**	***p*-value_FDR_**	**Fetal AUROC**	**Fetal *p*-value**
dopamine receptor signaling pathway	43	0.659	1	0.00908	0.575	0.0446
nucleotide-sugar metabolic process	37	0.665	1	0.0135	0.596	0.0217

#### Disease Associated Gene Set Enrichment

**FIGURE 3:**
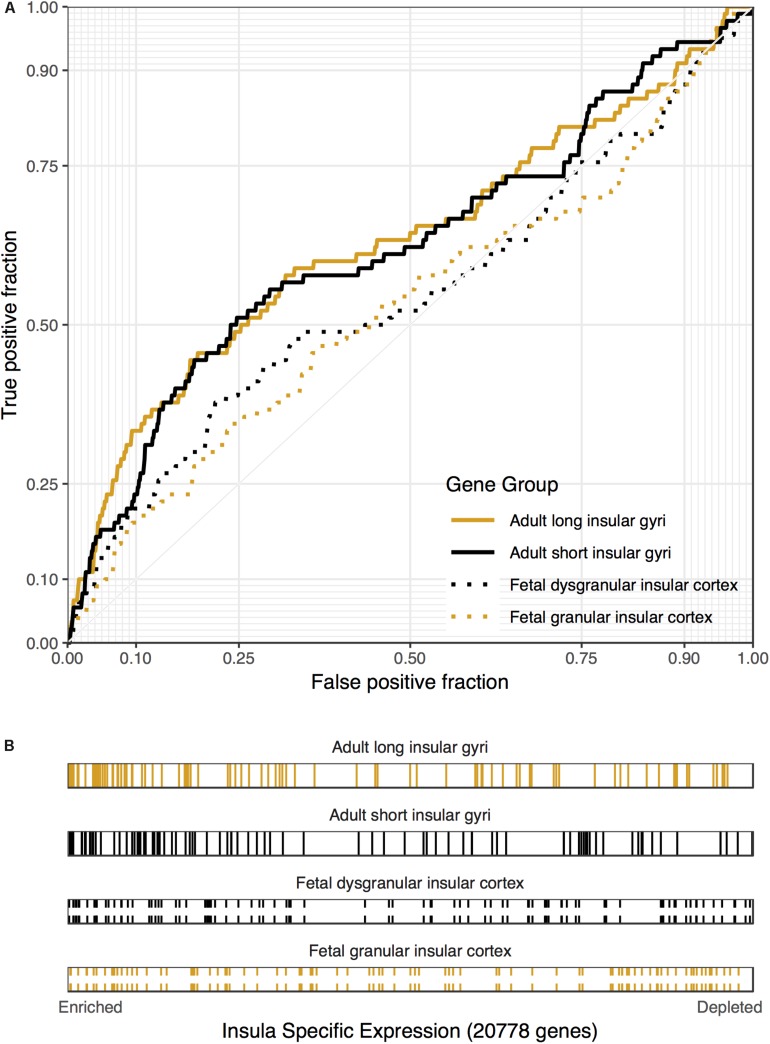
Associations between cocaine-related disorder genes and insular specific expression. **(A)** ROC curves showing the proportion of cocaine-related disorder genes that overlap (*y*-axis, true positive fraction) in varying lengths of the insular specific gene rankings (approximated by the *x*-axis, false positive fraction). **(B)** Distributions of the cocaine-related disorder genes across the insular specific gene rankings with each gene representing a single colored line. Color marks the short/dysgranular (black) and long/granular (orange) rankings. Dashed lines are used for the fetal datasets.

We first tested for enrichment across all 1,848 disease associated gene lists from the DisGeNET database (full results in Supplementary Tables S5, S6). Genes annotated to cocaine-related disorders was the top result in both regions (Figure 3). For the long insular gyri this was the only disease associated set that survives multiple test correction (90 genes, *p*_FDR_ = 0.014, AUROC = 0.63). For this result, five regions have a higher AUROC value for this set (specificity rank = 5). As detailed above, these genes are not enriched in the fetal granular IC but are in both the short insular gyri and the fetal dysgranular IC (Table 5). Genes associated with mood disorders were also significantly enriched in the short insular gyri. No other disease gene sets were significant after correcting for multiple comparisons in the adult data.

**TABLE 5:** Disease gene sets enriched in the adult short insular gyri after multiple test correction.

**Name**	**Genes**	**AUROC**	**Specificity Rank**	***p*-value_FDR_**	**fetal AUROC**	**fetal *P*-value**
Cocaine-related disorders	90	0.626	14	0.0315	0.557	0.031
Mood disorders	183	0.586	5	0.0315	0.510	0.318

#### Addiction Focused Gene Set Enrichment

Given past associations between the insula and addiction, we searched disease terms pertaining to addiction. As described above, cocaine-related disorders was the most significantly up-regulated disease association in both the long and short insular gyri. This was also significant in the fetal dysgranular IC (90 genes, *p*_uncorrected_ < 0.032, AUROC = 0.56, specificity rank = 73 out of 283 regions). Genes linked to amphetamine-related disorders were expressed at above average levels in the adult long and short insular gyri (74 genes, *p*_uncorrected_ < 0.020, AUROC > 0.56, specificity rank < 7), as well as in the fetal granular IC (*p*_uncorrected_ < 0.022, AUROC > 0.56, specificity rank = 37). Substance-related disorders were also expressed at higher levels in the adult long insular gyri and fetal granular and dysgranular IC (111 genes, *p*_uncorrected_ < 0.040, AUROC > 0.54, specificity rank < 36). Substance withdrawal syndrome associated genes were enriched in the adult long and short insular gyri (53 genes, *p*_uncorrected_ < 10^–3^, AUROC > 0.62, specificity rank < 12). Beyond the cocaine-associated genes, these findings would not survive correction for the 1,848 disease associated gene lists tested and enrichment was not found for terms relating to marijuana, alcohol, or tobacco.

We also examined GO terms pertaining to drugs of abuse. Both the adult long and short insular gyri and the fetal dysgranular and granular IC had enriched expression of genes annotated to the behavioral response to cocaine term (19 genes, *p*_uncorrected_ < 0.018, AUROC > 0.63, specificity rank < 39). “Response to cocaine” was also enriched in the fetal granular IC (51 genes, *p*_uncorrected_ < 0.026, AUROC = 0.58, specificity rank = 22). In the adult data, “response to morphine” was enriched (32 genes, *p*_uncorrected_ < 0.0074, AUROC > 0.62, specificity rank < 15) in the long and short insular gyri. This enrichment was significant in the short insular gyri when correcting for the number of GO groups tested (*p*_FDR_ < 0.02). In the fetal data, “secondary alcohol biosynthetic process” was enriched in the dysgranular and granular IC (69 genes, *p*_uncorrected_ < 0.032, AUROC > 0.56, specificity rank < 45). “Alcohol binding” was also enriched in the fetal dysgranular IC (79 genes, *p*_uncorrected_ < 0.029, AUROC = 0.56, specificity rank = 28). No enrichment was found for nicotine associated gene sets.

Next, we tested genes from genome-wide association studies of addiction. These included studies relating to opioids, smoking, alcohol, and cannabis. None were significantly enriched, except for seven genes that are nearby genetic variants that have been associated with cannabis use. These seven are significantly enriched in both the fetal granular (*p*_uncorrected_ < 0.02, AUROC = 0.73, specificity rank = 14) and dysgranular IC (*p*_uncorrected_ < 0.01, AUROC = 0.77, specificity rank = 6) but not in the adult insula.

#### Depression Focused Gene Set Enrichment

In the DisGeNET database, no gene sets relating to depression were significantly enriched in the adult data nor the fetal data. However, genes associated with seasonal affective disorder were enriched in the adult long and short insular gyri (16 genes, *p*_uncorrected_ < 0.041, AUROC > 0.62, specificity rank < 33). The adult long insular gyri was also enriched for higher expression of genes linked to major affective disorder (21 genes, *p*_uncorrected_ < 0.024, AUROC = 0.63, specificity rank = 3) and anhedonia (27 genes, *p*_uncorrected_ < 0.0041, AUROC = 0.65, specificity rank = 7). We also tested genes from genome-wide association studies of depression. In the adult data, 53 genes associated with major depressive disorder were up-regulated (*p*_uncorrected_ < 0.037, AUROC > 0.57). The 257 genes from the gene based genetic analyses of depression were also enriched in the long insular gyri (*p*_uncorrected_ < 0.030, AUROC = 0.53). However, we note that this finding was not specific, as over 41 regions had higher AUROC values. In the fetal data, the 53 genes genetically associated with major depressive disorder were specifically enriched in the fetal dysgranular IC (53 genes, *p*_uncorrected_ < 0.003, AUROC = 0.61, specificity rank = 3). Overall, we do not observe a clear enrichment of expression for genes associated with depression in the insular region.

### Claustrum

#### Adult and Fetal Claustrum

**FIGURE 4:**
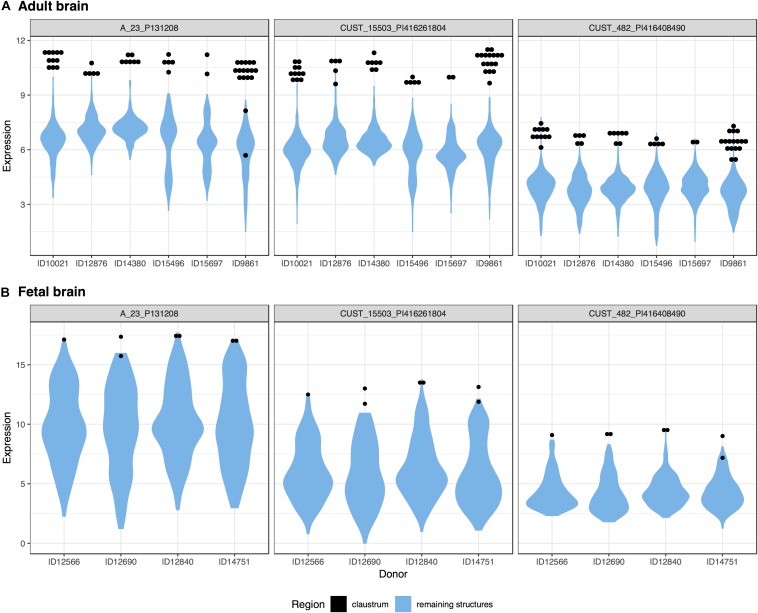
Plots of *NR4A2* expression in the adult **(A)** and fetal **(B)** brains. Expression (log2 intensity) is plotted on the *y*-axis for each of the three probes for *NR4A2*. Donor identification numbers are marked on the *x*-axis. Expression in the claustrum is marked in black, expression across the remaining brain regions is shown in blue violin plots.

We investigated the genes with significant up-regulation in the adult claustrum. Of the 20,778 tested genes, over 46% were significantly up-regulated in the 47 claustrum samples (9,591 genes, *p*_FDR_ < 0.05). The top 20 genes are presented in Table 6 with the full table in Supplementary Table S7. In the seven fetal claustrum samples, only 379 genes were significantly up-regulated (*p*_FDR_ < 0.05). Between the adult and fetal data of up-regulated genes, 261 genes intersect (hypergeometric test, *p* < 10^–18^), 3 of these are within the top 20 lists (*NR4A2, SMIM32*, and *GNB4*). The top-ranked gene, *NR4A2* (ranked 7th in the fetal data), is plotted in Figure 4.

**TABLE 6:** Top 20 enriched genes in the adult claustrum.

**Gene**	**Name**	**Significant**	***p*-value**
		**probes**	
NR4A2	Nuclear receptor subfamily 4 group A member 2	3	4.98E-209
SLC17A8	Solute carrier family 17 member 8	3	1.65E-205
ANXA1	Annexin A1	1	8.45E-187
NTNG2	Netrin G2	3	2.85E-180
RGS12	Regulator of G protein signaling 12	3	2.01E-179
UCMA	Upper zone of growth plate and cartilage matrix associated	2	3.46E-179
SMIM32	Small integral membrane protein 32	2	1.66E-160
STT3B	STT3B, catalytic subunit of the oligosaccharyltransferase complex	2	1.45E-157
GNB4	G protein subunit beta 4	4	5.54E-155
DCSTAMP	Dendrocyte expressed seven transmembrane protein	2	4.52E-152
L1TD1	LINE1 type transposase domain containing 1	2	2.94E-148
GPD2	Glycerol-3-phosphate dehydrogenase 2	2	7.76E-137
SMYD1	SET and MYND domain containing 1	2	1.61E-129
SPATA19	Spermatogenesis associated 19	2	2.21E-129
TPMT	Thiopurine *S*-methyltransferase	3	1.62E-125
THTPA	Thiamine triphosphatase	2	3.07E-121
C1QL4	Complement C1q like 4	1	2.40E-120
STK32B	Serine/threonine kinase 32B	2	2.47E-120
LAIR2	Leukocyte associated immunoglobulin like receptor 2	2	5.60E-119
PLA2G4A	Phospholipase A2 group IVA	3	4.89E-117

### Gene Ontology Enrichment

In the adult data, 495 GO groups were significantly enriched after correcting for multiple comparisons and 310 groups in the fetal data (*p*_FDR_ < 0.05, full results in Supplementary Table S8). The top result in the adult claustrum was synaptic vesicle cycle with 194 genes (*p*_FDR_ < 10^–8^, AUROC > 0.65). For the fetal claustrum, the top GO group was intrinsic component of synaptic membrane (166 genes, *p*_FDR_ < 10^–9^, AUROC > 0.66).

In addition, we filtered the results to only include groups that have a specificity rank of ≤ 1. After this cutoff, 159 groups survived in the adult claustrum and 61 groups in the fetal claustrum. Of the 159 significant and specific GO groups in the adult claustrum, 56 were also found in the fetal claustrum (*p*_uncorrected_ < 0.05). The top 20 are presented in Table 7.

**TABLE 7:** Top 20 specifically enriched GO groups in the adult claustrum that are also enriched in the fetal claustrum.

**Name**	**Genes**	**AUROC**	**Specificity rank**	***p*-value_FDR_**	**Fetal AUROC**	**Fetal *p*-value**
Regulation of macroautophagy	158	0.645	0	3.57E-07	0.623	4.48E-08
Cytosolic transport	151	0.641	0	9.03E-07	0.54	0.0456
Regulation of dendritic spine development	65	0.694	0	1.36E-05	0.564	0.0384
Vacuolar transport	127	0.637	1	1.98E-05	0.614	4.46E-06
Regulation of postsynaptic membrane neurotransmitter receptor levels	65	0.688	0	2.91E-05	0.674	6.06E-07
Protein serine/threonine phosphatase complex	48	0.712	0	4.89E-05	0.598	0.00923
Regulation of phosphatase activity	170	0.613	0	4.97E-05	0.56	0.00352
Clathrin-dependent endocytosis	40	0.725	0	9.02E-05	0.692	1.28E-05
Cytoskeleton-dependent intracellular transport	165	0.611	0	9.17E-05	0.542	0.0308
Regulation of phosphoprotein phosphatase activity	113	0.633	0	9.17E-05	0.582	0.00135
Regulation of protein dephosphorylation	134	0.619	1	1.71E-04	0.581	6.37E-04
Axo-dendritic transport	63	0.668	0	2.41E-04	0.582	0.0119
Autophagosome assembly	89	0.638	1	3.76E-04	0.635	5.53E-06
Clathrin vesicle coat	28	0.741	0	4.86E-04	0.7	1.25E-04
Vesicle coat	57	0.668	0	5.20E-04	0.601	0.00409
Organelle transport along microtubule	74	0.646	1	5.92E-04	0.627	8.24E-05
Membrane coat	99	0.622	1	9.25E-04	0.617	2.88E-05
Clathrin coat of coated pit	20	0.768	0	0.00117	0.712	5.28E-04
Vesicle docking	66	0.647	0	0.00118	0.606	0.00143
Vesicle-mediated transport to the plasma membrane	83	0.629	0	0.00143	0.6	8.44E-04

#### Disease Associated Gene Enrichment

Of the 1,848 disease gene sets from the DisGeNET database, four are significantly up-regulated in the adult claustrum (*p*_FDR_ < 0.05, Table 8, full results in Supplementary Table S9). These four are also specific with at most four regions having a higher AUROC value (of 232 regions). Severe intellectual disability (I.Q. 20–34) and epileptic encephalopathy were significantly enriched in both adult and fetal data.

**TABLE 8:** Disease gene sets enriched in the adult claustrum after multiple test correction.

**Name**	**Genes**	**AUROC**	**Specificity rank**	***p*-value_FDR_**	**Fetal AUROC**	**Fetal *P*-value**
HIV Infections	99	0.633	2	0.00547	0.476	0.798
Epileptic encephalopathy	26	0.731	3	0.0209	0.648	0.005
Severe intellectual disability (I.Q. 20-34)	99	0.616	4	0.0237	0.628	6.16E-06
Global developmental delay, severe	47	0.661	2	0.0333	0.475	0.724

#### Addiction Focused Gene Set Enrichment

We examined disease terms relating to addiction using the DisGeNET database. Similar to the insula analyses, in the adult data claustrum, genes associated with “cocaine-related disorders” were enriched (90 genes, *p*_uncorrected_ < 0.0072, AUROC = 0.58, specificity rank = 79 out of 232 regions). This was also significant in the fetal data (90 genes, *p*_uncorrected_ < 0.0027, AUROC = 0.59, specificity rank = 31 out of 283 regions). Also, in the fetal data, substance withdrawal syndrome (53 genes, *p*_uncorrected_ < 0.0021, AUROC = 0.61, specificity rank = 26), amphetamine-related disorders (74 genes, *p*_uncorrected_ < 0.0024, AUROC = 0.60, specificity rank = 10), psychoses (substance-induced) (17 genes, *p*_uncorrected_ < 0.013, AUROC = 0.66, specificity rank = 11) and alcohol abuse (44 genes, *p*_uncorrected_ < 0.044, AUROC = 0.58, specificity rank = 87) were enriched. No significant enrichment was found for terms relating to marijuana or tobacco.

GO terms pertaining to drugs of abuse were also searched. In the adult data, response to morphine (32 genes, *p*_uncorrected_ < 0.016, AUROC = 0.61, specificity rank = 22), regulation of alcohol biosynthetic process (73 genes, *p*_uncorrected_ < 0.022, AUROC = 0.57, specificity rank = 42), and alcohol biosynthetic process (148 genes, *p*_uncorrected_ < 0.041, AUROC = 0.54, specificity rank = 70) were significantly enriched. While in the fetal data we found behavioral response to cocaine (19 genes, *p*_FDR_ < 0.019, AUROC = 0.72, specificity rank = 3) to be significantly enriched after multiple test correction. No significant enrichment was found for terms relating to nicotine.

In addition, three genes genetically associated with ever smoking were enriched in the adult claustrum (*BDNF*, *APBB2*, and *CDC27*; *p*_uncorrected_ < 0.02, AUROC = 0.84, specificity rank = 1). In the fetal claustrum, two genes associated with smoking cessation also rank high (*SLC25A21* and *SEMA6D*; *p*_uncorrected_ < 0.048, AUROC = 0.84, specificity rank = 45).

#### Depression Focused Gene Set Enrichment

Depression associated gene sets in the DisGeNET database were also investigated for significant enrichment in the claustrum. In the adult data, we found seasonal affective disorder to be enriched (16 genes, *p*_uncorrected_ < 0.046, AUROC = 0.62, specificity rank = 37). This was also found in the fetal data (*p*_uncorrected_ < 0.0042, AUROC = 0.69, specificity rank = 9). In fetal data, genes annotated to “anhedonia” were significantly enriched, with no other regions having a higher AUROC value (27 genes, *p*_FDR_ < 0.048, AUROC = 0.71, specificity rank = 0). Also, genes associated with drug-induced depressive state (14 genes, *p*_uncorrected_ < 0.033, AUROC = 0.64, specificity rank = 27) and depression (bipolar) (12 genes, *p*_uncorrected_ < 0.035, AUROC = 0.65, specificity rank = 58) were strongly expressed in the fetal claustrum. Enrichment of genes identified in large genetic studies of depression (257 genes, *p*_uncorrected_ < 0.0005, AUROC = 0.56, specificity rank = 12) and major depressive disorder (53 genes, *p*_uncorrected_ < 0.005, AUROC = 0.61, specificity rank = 5) are found in the adult claustrum but not in the fetal samples. Overall, expression of depression associated genes appear to be higher in the claustrum than the insular regions.

### Cell Type Proportions

Based on the adult transcriptomic data and marker genes, we estimated proportions of neurons, oligodendrocytes, oligodendrocyte precursors, microglia and endothelial cell-types (Supplementary Table S10). Relative to all other regions, neurons have the highest relative proportion estimates in the insular-claustrum region. Specifically, only five other regions have a higher estimated amount of neurons, while the short and long insular gyri rank 11th and 28, respectively (of 232 regions). The claustrum also has a high estimated proportion of oligodendrocytes (specificity rank of 49). For the remaining cell-types, we do not observe notable proportion estimates (specificity ranks < 81).

### Transcriptomic Similarities Between the Claustral and Insular Regions

**FIGURE 5:**
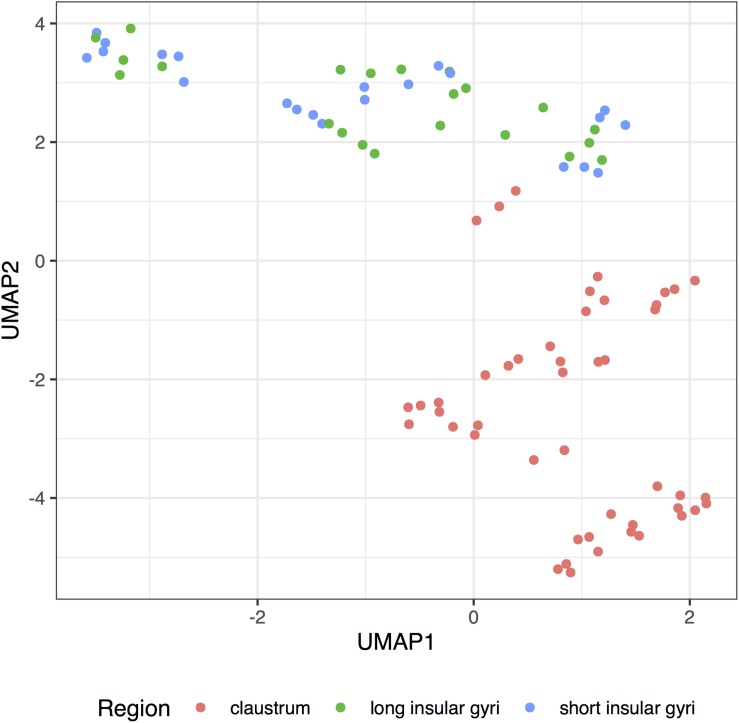
UMAP projection of the insular and clastral samples. Complete transcriptome profiles are reduced to a two-dimensional space (UMAP1 and 2) for visualization. Each dot represents a sample with color marking the sampled region (claustrum: red; long insular gyri: green; long insular gyri: blue).

We reduced the dimension of the adult claustral and insular samples to obtain a transcriptome-wide visualization of the similarities between the regions. Using the complete expression profiles, we observe that the claustral samples can be separated from the insular profiles (Figure 5). Within the insula, we do not see a clear grouping of samples from the long and short gyri. While the claustral samples are central in the fetal data, we did not find apparent regional clustering (Supplementary Figure S1).

We next computed the transcriptional similarity between the adult claustrum and all other regions. We calculated which adult and fetal brain regions have enriched expression of the 20 most specific genes in the adult claustrum to determine which brain regions strongly express the adult claustrum markers (Table 9). Brain-wide, insular regions have the highest expression of the adult claustrum marker genes. Of the 232 adult regions tested, 38 are significantly enriched for the marker genes (*p*_FDR_ < 0.05, AUROC > 0.5). Of those, 36 are neocortical regions, and the remaining two are the piriform cortex (paleocortex) and parahippocampal gyrus bank of the cos (transitionary). Across all regions, the neocortex is enriched for these marker genes (80 regions, mean AUROC = 0.642). In the adult data, the short gyri is more similar than the long insular gyri. Interestingly, in the fetal results, the claustrum is ranked lower than samples from the insular cortex. The lower absolute AUROC values in the fetal data are likely due to global expression differences between the fetal and adult data. Of the six zones assayed in the insular cortex, the intermediate zone, which is the deepest, has the most specific expression of the 20 claustral markers. We also note that the three-layered piriform cortex has high expression of the markers in both the adult and fetal data. Taken together, we found that the expression of claustral markers is highest in the insula and neocortex.

**TABLE 9:** The top ten fetal and adult regions that specifically express the 20 most up-regulated genes in the adult claustrum.

**Rank**	**Adult region**	**AUROC**	**Fetal region**	**AUROC**
		**Adult**		**Fetal**
1	Claustrum	1	Intermediate zone in granular insular cortex	0.783
2	Short insular gyri	0.94	Layer III of piriform cortex	0.764
3	Long insular gyri	0.905	Intermediate zone in dysgranular insular cortex	0.756
4	Frontal operculum	0.888	Claustrum	0.719
5	Planum polare	0.856	Subplate zone in dysgranular insular cortex	0.706
6	Posterior orbital gyrus	0.842	Intermediate zone in primary auditory cortex	0.703
7	Temporal pole, inferior aspect	0.842	Midbrain reticular formation	0.703
8	Temporal pole, superior aspect	0.82	Molecular layer of caudal subiculum	0.691
9	Temporal pole, medial aspect	0.809	Subplate zone in caudal perirhinal cortex	0.687
10	Piriform cortex	0.771	Posterior hypothalamic nucleus	0.673

## Discussion

In this study, we defined the adult and fetal insular cortex and claustrum at the molecular level. We identified gene expression enrichment in these areas concerning diseases, addiction, depression, and function. With regards to the insula, we found genes associated with addiction, most notably cocaine, as well as depression, mood disorders, glutamate and dopamine signaling, learning, memory, cardiac muscle contraction, and oxygen transport. In the claustrum, we found that genes associated with addiction, depression, human immunodeficiency virus (HIV), severe intellectual disability, seizures, epilepsy, intracellular transport, spine development, and macroautophagy had enriched expression. Across the brain, we found that the insula has the highest transcriptomic similarity to the claustrum.

### Top Ranked Genes

In terms of individual genes, we found *UCMA* to be the most significantly enriched gene in the adult long and short insular gyri. It was also present in the adult claustrum (ranked 6th). While its function in the brain is unknown, the *UCMA* gene has been shown to mark a specific transcriptomic type of layer 5 neurons in a study of the mouse visual and motor cortices (Tasic et al., 2016).

Previous studies have identified specific claustral expression of *NR4A2*, *NTNG2*, *GNG2*, *OPRK1*, *CUX2*, and *LXN* in rodents and primates (Peckys and Landwehrmeyer, 1999; Mathur et al., 2009; Pirone et al., 2012; Watakabe et al., 2014). *NR4A2*, *NTNG2*, and *LXN* are in our top 20 lists and together, these six genes are strongly enriched in the adult and fetal claustrum (*p* < 0.0005, AUROC > 0.92). *NR4A2* and *NTNG2* were also present in the insula’s top 20 lists. *NR4A2*, also known as *NURR1*, is essential for the differentiation of dopaminergic neurons (Zetterström et al., 1997; Saucedo-Cardenas et al., 1998). It regulates gene expression of numerous factors that are important to the dopamine system; among these are dopamine transporter and tyrosine hydroxylase (Sakurada et al., 1999; Sacchetti et al., 2001). *NR4A2* has been implicated in drug addiction (Bannon et al., 2002; Horvath et al., 2007), Parkinson’s disease (Jankovic et al., 2005; Le et al., 2008; Zhang et al., 2012), schizophrenia and bipolar disorder (Buervenich et al., 2000; Xing et al., 2006). This is in line with the enrichments we found regarding addiction and depression (bipolar) gene sets in the IC and claustrum, as well as dopamine activity in the IC. Although we did not observe any gene sets indicating an association with Parkinson’s disease and schizophrenia, studies have implicated the IC and claustrum in these diseases (Kalaitzakis et al., 2009; Cascella et al., 2011; Chen et al., 2016; Criaud et al., 2016; Arrigo et al., 2018; Joutsa et al., 2018).

### Addiction

Genes associated with cocaine, amphetamine, morphine, alcohol and withdrawal were found to be significant in the IC. Cocaine seems to be the most prominently associated drug of abuse in the insula region as it was the only term that passed multiple test correction. The insula has long been known to play roles in addiction (Droutman et al., 2015b). One of the main studies credited for bringing attention to the insula found that stroke patients that suffered damage to the insula were more likely to quit smoking immediately after lesion onset compared to non-insular damage (Naqvi et al., 2007). Since then, numerous studies have linked the insula with having a prominent role in addictions. Preclinical studies have involved the insula in motivation, nicotine-taking and -seeking behaviors (Forget et al., 2010; Pushparaj et al., 2013), gambling (Pushparaj et al., 2015) and alcohol addiction (Pushparaj and Le Foll, 2015). Numerous clinical studies have also provided valuable information relating the insula and addiction. For instance, cocaine users were found to have decreased gray matter in the insula (Ersche et al., 2011; Gardini and Venneri, 2012), as well as greater connectivity within the salience network (i.e., the anterior IC and anterior cingulate cortex) (Wisner et al., 2013). It was unexpected that no enrichment was found for terms relating to nicotine since many preclinical and clinical studies found insular differences that were associated with nicotine dependence (Droutman et al., 2015b). Given that nicotine triggers the release of dopamine, we note that genes in the dopamine signaling pathway are strongly enriched in the adult and fetal insula, suggesting downstream relationships.

Dopamine is broadly associated with addiction, as all drugs of abuse increase dopamine levels (Nestler, 2005). It has been long known that dopamine is a key player in addiction [see our review (Le Foll et al., 2009)] and is involved in dopamine utilization (Gaspar et al., 1989). Our finding of enriched expression of genes in the dopamine signaling pathway is specific as only one other brain region was found to have a higher AUROC value. Of these 43 genes, dopamine receptors 1 (ranked 5th), 3 (14th), 4 (22nd) and 5 (8th) are ranked in the top half with *DRD2* having depleted expression (ranked second last). High expression of *DRD1* mirrors previous studies that found higher expression of *DRD1* and low expression of *DRD2* (Hurd et al., 2001). Functional studies have found insular infusions of a D_2_ antagonist did not have an effect on nicotine self-administration, but a D_1_ antagonist did (Kutlu et al., 2013). Although it is not as established as dopamine, glutamate has also been shown to play a role in addiction (Tzschentke and Schmidt, 2003; D’Souza, 2015). In the long insular gyri, three gene ontology groups relating to glutamate signaling and activity were enriched, all validated in the fetal data and had high specificity to the insula. Researchers have demonstrated that glutamate plays a role in drug seeking and reinstatement (Kalivas, 2009; Knackstedt and Kalivas, 2009). Drugs of abuse also alter the transmission of glutamate either by indirectly or directly acting on its receptors (D’Souza, 2015). Clinically, inhibition of glutamate release is a potential target for cocaine addiction treatment (Schmidt and Pierce, 2010; D’Souza, 2015; Caprioli et al., 2018).

### Depression

We find limited evidence of higher expression of depression associated genes in the insula and claustrum. However, genes associated with mood disorders are ranked as the second and third top disease gene sets associated with the adult short and long insular gyri, respectively (specificity <10). The literature supports the insula and claustrum as having some involvement with depression. Studies have shown that patients with depression have altered functional connectivity in the insula, specifically the anterior region (Veer et al., 2010; Kandilarova et al., 2018; Wang et al., 2018). Gray matter reductions in the anterior insula were also found in patients with major depressive disorder (Lee et al., 2011; Stratmann et al., 2014). A meta-analysis found the insula was consistently identified in imaging studies of depression across methods and study design (Fitzgerald et al., 2008). In comparison to the insula, stronger enrichment of expression for depression associated genes was found in the claustrum. Possibly due to its size and location, the claustrum has not been identified in imaging studies of depression. However, a postmortem study found bilaterally reduced claustral volumes in major depressive disorder (Bernstein et al., 2016). Anhedonia, one of the main symptoms in depression, has been linked to both the insula and claustrum in our results. The most specific depression associated result is the enrichment of “anhedonia” genes in the fetal claustrum. Anhedonia symptoms in individuals with unipolar or bipolar depression were negatively correlated with metabolism in the insula and claustrum (Dunn et al., 2002). Furthermore, in a healthy population of adolescents, those who scored higher on anhedonia measures exhibited decreased activation in the claustrum and insula compared to those who had lower anhedonia scores (Chan et al., 2016). Altogether, we highlight the relevance of this region to mood disorders and anhedonia while suggesting more attention be given to the claustrum.

### Learning and Memory

In the long insular gyri and granular IC, we found a set of 141 genes associated with learning to be significantly and specifically enriched. Although not validated in the fetal data, associative learning was enriched in the adult data. In addition, a memory gene set was enriched in the long insular gyri, with only two other brain regions holding a higher AUROC value. At first glance, the enrichment may be explained by the IC role in taste learning. Studies have indicated its role in working memory of taste (Ragozzino and Kesner, 1999), conditioned taste aversion (Schier et al., 2016) and taste learning [see review by Yiannakas and Rosenblum (2017)]. This is in part mediated by glutamatergic and dopaminergic transmission (Guzmán-Ramos et al., 2010; Osorio-Gómez et al., 2017), which also showed enrichment in the insula. However, no gene ontology groups corresponding to taste specifically were enriched in the IC. Although this was surprising, the insula has been implicated in other areas of learning and memory, which may explain these gene set enrichments. For instance, the insula is activated during a learning/memory task of human face recognition (Paller et al., 2003) and object recognition memory (Bermudez-Rattoni et al., 2005). While the long insular gyri has an above average estimated proportion of neurons, the short gyri and claustrum have higher estimates, suggesting this signal is not due to a high proportion of neurons. However, a single-cell dissection of the insula may highlight specific neuron types.

### Cardiac Muscle Contraction

One interesting finding from the gene ontology enrichment in the IC was that of cardiac muscle contraction. We found a gene set of 127 genes relating to cardiac muscle contraction to be significantly and specifically enriched in the long insular gyri. Broadly, the insula has been described as a key region in the brain-heart axis (Nagai et al., 2010). Introception, and specifically awareness of one’s heartbeat is among the bodily states that the insula is believed to mediate (Craig, 2002, 2003). This has been shown using heartbeat monitoring tasks whereby researchers have found the insula to be activated (Pollatos et al., 2007; Zaki et al., 2012; Kuehn et al., 2016). These findings are mainly found in the anterior insula. However, the enrichment we observed was in the posterior region. This may be due to the fact that the posterior insula is the receiver of interoceptive signals, which is then sent to the anterior insula (Craig, 2002). Furthermore, damage to the insula was associated with electrocardiographic abnormalities (Abboud et al., 2006) and an increase in adverse cardiac outcomes (e.g., myocardial infarction, new-onset angina, sudden cardiac death) (Laowattana et al., 2006). While these neural connections between the insula and heart are interesting, it’s not clear why genes that function in muscle contraction are enriched in the insula.

### Oxygen Transport

We found high and specific enrichment of two oxygen-related GO terms, oxygen transport and oxygen carrier activity, in the adult long insular gyri. This enrichment, was validated in the fetal dysgranular IC as well. It is not known why these genes are enriched here, but we suspect that it could be due to the insula’s role in homeostasis. Similarly to the implication of the insula in regulating heartbeat described above, it has also been implicated in regulating breathing and respiration (Kaada and Jasper, 1952; Showers and Lauer, 1961; Hassanpour et al., 2018). In addition, dyspnea (i.e., breathlessness) has been shown to activate the insula (Banzett et al., 2000; von Leupoldt et al., 2008; Esser et al., 2017). However, these studies have found other areas to also be involved; therefore, further studies are needed to clarify why these gene sets were specifically enriched in the insula.

### Epilepsy

Genes associated with seizures and epilepsy were enriched in the claustrum. Specifically, genes linked to epileptic encephalopathy, a severe and early onset disorder are strongly enriched. An example gene is *GNAO1*, which is the 23rd most specifically expressed gene in the adult claustrum and is known to cause early onset epileptic encephalopathy (Nakamura et al., 2013; Law et al., 2015). Researchers have found that the claustrum was involved early on in kainate-induced seizures and in some instances, the seizure originated in the claustrum (Bayat et al., 2018). A number of studies using magnetic resonance imaging (MRI) have found a link between the claustrum and epilepsy (Sperner et al., 1996; Nixon et al., 2001; Ishii et al., 2011; Meletti et al., 2015, 2017). Although not all seizures affect consciousness, it remains an important feature of seizures (Blumenfeld, 2012). As we previously mentioned, the exact role of the claustrum is not well understood, however, many theories focus around the idea of its involvement in consciousness (Crick and Koch, 2005; Chau et al., 2015; Yin et al., 2016). A case study of an epileptic patient reported that stimulating an electrode placed between the claustrum and anterior IC disrupted consciousness, which was then regained after stimulation stopped (Koubeissi et al., 2014). In fact, the role of the claustrum in consciousness during seizures has been the subject of a recent review (Kurada et al., 2019). Thus, given the enrichment of epilepsy-related genes and the link between consciousness and seizures, this may further allude to the claustrum’s role in consciousness.

### Human Immunodeficiency Virus

Genes associated with HIV infections were enriched and had high specificity to the claustrum. In support of this, researchers have found astrogliosis (Sevigny et al., 2005) and increased fractalkine (Tong et al., 2000) in the claustrum of people with HIV encephalitis. Fractalkine is a chemokine encoded by the *CX3CL1* gene, which we found to be significantly enriched in both the adult and fetal claustrum. Furthermore, a study on postmortem brains of children who died of acquired immunodeficiency syndrome (AIDS) found the claustrum, among other brain regions, to have a decreased volume (Kozlowski et al., 1997).

### Intracellular Transport

Genes that function in intracellular transport are expressed at higher levels in the claustrum. Specifically enriched GO terms include cytosolic, vacuolar, cytoskeleton-dependent, and axo-dendritic transport. White matter has long been considered to be a transport system (Paus et al., 2014). Our findings of increased axo-dendritic transport related genes, combined with the region’s wide-ranging projections (Torgerson et al., 2015; Wang et al., 2017) suggest that the claustrum may be a key hub in this transport network.

### Macroautophagy

The most specific GO group for the claustrum was “regulation of macroautophagy”. We were unable to find any reports in the literature of enhanced macroautophagy or related genes in the claustrum. Given its location in between white matter tracts, we speculate the claustrum may undertake increased macroautophagy based myelin remodeling. Such autophagic mechanisms have shown to be important in the peripheral nerve injury and amyotrophic lateral sclerosis but have not been linked to the claustrum (Ceballos-Diaz et al., 2015; Gomez-Sanchez et al., 2015; Qin et al., 2018). Like the long motor neurons that degenerate in amyotrophic lateral sclerosis, we suspect that a high level of autophagy might be needed to support the connectivity of the claustrum (Torgerson et al., 2015).

### Insula-Claustrum Comparisons

In a brain-wide analysis, we found that the insula has the highest expression of the claustral markers. More broadly, claustral specific genes are enriched in neocortical regions. Surprisingly, the intermediate zone of the developing insular cortex had higher expression of these genes than the fetal claustrum samples. More specifically, of all the fetal regions, the intermediate zone of the insula had the highest expression of the adult claustral markers. This zone is the deepest of those assayed and thus closest to the insula. The strong expression of adult claustral markers in this zone may inform future developmental studies. Overall, these findings reinforce rodent studies that found the claustrum and insula have a strong ontogenetic relationship through a shared lateral pallium origin (Puelles, 2014; Watson and Puelles, 2017; Binks et al., 2019).

### Strengths and Limitations

This study benefits from two brain- and genome-wide Atlases of the human brain. This coverage allowed us to transfer information from anatomy to genes. By using two transcriptomic Atlases, we were able to test signals obtained from the adult in the fetal brain, providing reproducibility across development. Although this approach reveals novel molecular features of the insula and claustrum, there are some limitations. First, the sample size is limited (six adult brains and four fetal brains). Second, sex is not balanced with five male adult brains and only one female adult brain. The reverse issue is present in the fetal brains, whereby three are female. However, we note that the number of genes differentially expressed across regions dwarfs the number that are sex or even species specific (Strand et al., 2007; Toker et al., 2016). We also note that we use gene expression profiles of bulk tissue, which contain variable proportions of cell-types. Differences in cell-type proportions may be the primary drivers of the extracted signals and not the differential expression of specific genes. At the coarse level, our analysis of estimated cell-type proportions did not suggest that our findings are primarily driven primarily by proportion differences.

## Conclusion

In conclusion, our study of insular and claustrum specific gene expression links these regions to an array of functions and diseases. Many of these gene enrichments were expected, such as that of genes associated with addiction, as well as some of the top individual genes found in the claustrum, which are known to be claustral markers. Our finding of enrichment of epilepsy gene sets in the claustrum could further allude to its role in consciousness, which has been hypothesized to be its primary function. Insular associations with oxygen transport and cardiac muscle contraction molecules reinforce it’s past links to interoceptive awareness. Combining the results, we find associations with learning, memory, severe intellectual disability, and epilepsy, suggesting the insula-claustrum region plays critical roles in cognition. Other findings, such as the enrichment of genes involved HIV and macroautophagy in the claustrum require further investigation. In addition, our findings of strong transcriptomic similarity between the two regions confirm their ontogenetic relationship. Altogether, our results provide a novel molecular perspective on the unique properties of the insula-claustrum region.

## Data Availability Statement

Publicly available datasets were analyzed in this study. This data can be found at http://www.brain-map.org and http://www.brainspan.org.

## Author Contributions

BL and LF: conception and design. LF: analysis of data. CI, BL, and LF: interpretation of data. CI: writing the first draft of the manuscript. BL and LF: critical revision of the manuscript.

## Conflict of Interest

The authors declare that the research was conducted in the absence of any commercial or financial relationships that could be construed as a potential conflict of interest.

## Footnotes

1 http://help.brain-map.org/display/humanbrain/Documentation

2 http://help.brain-map.org/display/devhumanbrain/Documentation

## Supplementary Material

Scripts, supplementary tables, and data files for reproducing the analyses are available online at https://github.com/leonfrench/InsulaClaustrumSpecific and https://figshare.com/articles/Trans criptomic_Characterization_of_the_Human_Insular_Cortex_and_Claustrum/8044727.
